# Associations between subregional thalamic volume and brain pathology in autosomal dominant Alzheimer’s disease

**DOI:** 10.1093/braincomms/fcab101

**Published:** 2021-05-10

**Authors:** Enmanuelle Pardilla-Delgado, Heirangi Torrico-Teave, Justin S Sanchez, Liliana A Ramirez-Gomez, Ana Baena, Yamile Bocanegra, Clara Vila-Castelar, Joshua T Fox-Fuller, Edmarie Guzmán-Vélez, Jairo Martínez, Sergio Alvarez, Martin Ochoa-Escudero, Francisco Lopera, Yakeel T Quiroz

**Affiliations:** 1 Massachusetts General Hospital, Harvard Medical School, Boston, MA 02114, USA; 2 Grupo de Neurociencias de Antioquia, Universidad de Antioquia, Medellin 050010, Colombia; 3 Boston University, Boston, MA 02215, USA; 4 Hospital Pablo Tobon Uribe, Medellin 050034, Colombia

**Keywords:** presenilin-1, thalamus, MRI, PET imaging, preclinical

## Abstract

Histopathological reports suggest that subregions of the thalamus, which regulates multiple physiological and cognitive processes, are not uniformly affected by Alzheimer’s disease. Despite this, structural neuroimaging studies often consider the thalamus as a single region. Identification of *in vivo* Alzheimer’s-dependent volumetric changes in thalamic subregions may aid the characterization of early nuclei-specific neurodegeneration in Alzheimer’s disease. Here, we leveraged access to the largest single-mutation cohort of autosomal-dominant Alzheimer’s disease to test whether cross-sectional abnormalities in subregional thalamic volumes are evident in non-demented mutation carriers (*n* = 31), compared to non-carriers (*n* = 36), and whether subregional thalamic volume is associated with age, markers of brain pathology and cognitive performance. Using automatic parcellation we examined the thalamus in six subregions (anterior, lateral, ventral, intralaminar, medial, and posterior) and their relation to age and brain pathology (amyloid and tau), as measured by PET imaging. No between-group differences were observed in the volume of the thalamic subregions. In carriers, lower volume in the medial subregion was related to increased cortical amyloid and entorhinal tau burden. These findings suggest that thalamic Alzheimer’s-related volumetric reductions are not uniform even in preclinical and prodromal stages of autosomal-dominant Alzheimer’s disease and therefore, this structure should not be considered as a single, unitary structure in Alzheimer’s disease research.

## Teaser

Pardilla-Delgado et al. studied individuals with autosomal-dominant Alzheimer’s disease and found that lower medial and posterior thalamic subregional volumes were associated with greater amyloid and tau burden, years before clinical onset. Findings suggest that studying thalamic sub-regions could improve our understanding of the role of this structure in Alzheimer’s disease.

## Graphical Abstract

**Graphical Abstract:**
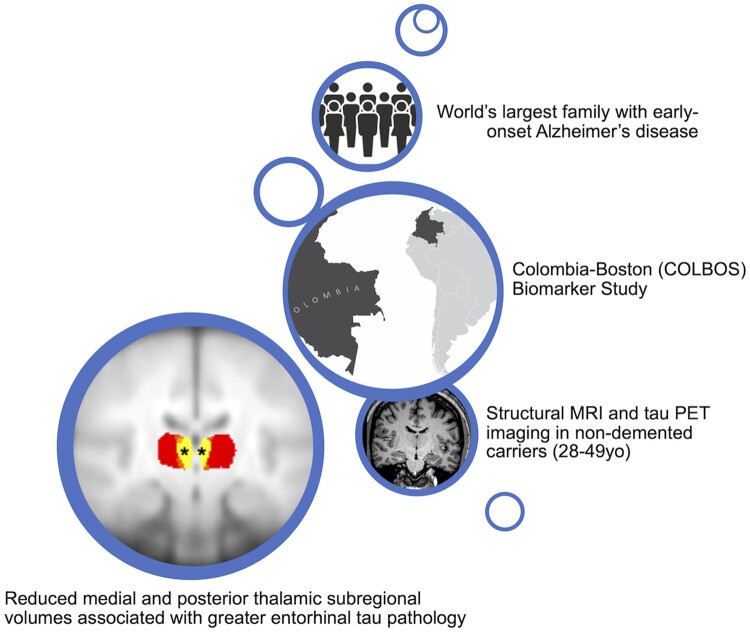

## Introduction

Research in preclinical Alzheimer’s disease, when individuals may have Alzheimer’s disease-related pathology in the absence of cognitive impairment, has focused on identifying biomarkers that can improve early detection of Alzheimer’s disease in individuals at increased risk.^1^ Current biomarkers include amyloid-beta and tau pathology, as measured by PET and CSF, as well as hippocampal atrophy.^1^ The thalamus is the main relay of sensorimotor information in the brain and is thought to be crucial for memory processing^2^^,^^3^ and sleep-wake regulation, which are known to be impacted early in Alzheimer’s disease.^4^ Postmortem studies also have shown that Alzheimer’s disease-related pathology can be seen in specific thalamic nuclei early in the disease, i.e. preclinical Braak stages I–II.^5^^,^^6^ Despite its importance in early Alzheimer’s disease, the thalamus has not been included in structural MRI-based biomarkers of the disease, which are limited to subcortical regions within the temporal lobe.^7^^,^^8^

It remains unclear whether thalamic neurodegeneration, as measured by structural MRI, is evident during preclinical and prodromal stages of Alzheimer’s disease. MRI studies have shown altered anatomical and functional connectivity in thalamic regions in individuals with mild cognitive impairment (MCI) and Alzheimer’s disease.^9^^,^^10^ Research examining cognitively healthy *APOE4* carriers, with increased genetic risk for Alzheimer’s disease, has resulted in mixed findings. Reports show no thalamic volumetric reduction in healthy young^11^ or older adult *APOE4* carriers,^12^ while others have found a dose-response effect, with E4 homozygotes having the lowest thalamic volume.^13^

Autosomal dominant Alzheimer’s disease (ADAD) provides a unique opportunity to study biomarker changes during preclinical Alzheimer’s disease, as mutation carriers are virtually guaranteed to develop Alzheimer’s disease. Research with relatively small and heterogenous ADAD samples suggests that thalamic volume is reduced in asymptomatic mutation carriers compared to non-carriers, even in the absence of temporal lobe atrophy.^14^^,^^15^ In larger samples with ADAD due to different mutations, thalamic volume was lower in carriers relative to non-carriers years before the estimated age of symptom onset.^16^^,^^17^ Our group, which follows the largest cohort of ADAD due to a single mutation (*PSEN1* E280A), has found similar results in this homogeneous sample, i.e. reduced thalamic volume in asymptomatic carriers.^18^ However, others have reported reduced thalamus volume only after the onset of cognitive impairment in ADAD mutation carriers.^19^

The findings presented so far are constrained by evaluating the thalamus as a single, homogenous structure. To our knowledge, only one study has investigated volumetric changes in thalamic subregions in the prodromal Alzheimer’s disease continuum,^20^ which showed no differences in subregions between healthy older adults and participants in the prodromal Alzheimer’s disease stages (i.e. amyloid-positive MCI). Alzheimer’s disease patients had reduced volumes in anterior and posterior subregions compared to MCI and controls. These conflicting results, particularly in preclinical sporadic Alzheimer’s disease, reinforce the importance of assessing the thalamus as a complex, multi-nuclei structure.

In this study, we investigated whether volume of thalamic subregions is reduced in ADAD by studying non-demented carriers with the *PSEN1* E280A mutation from the Colombian kindred. Cognitive decline and pathological progression in this kindred have been well characterized,^21^^,^^22^ with an onset of MCI at median age 44 and dementia at age 49, allowing the use of carriers’ age as a proxy for longitudinal change in cross-sectional samples. We assessed whether the volume of thalamic subregions was associated with age, markers of *in vivo* brain pathology (PET amyloid-beta and tau burden), and cognitive performance.

## Materials and methods

### Participants

A total of 67 participants were recruited from the Alzheimer’s Prevention Initiative as part of the Colombia-Boston (COLBOS) biomarker study, in collaboration with the University of Antioquia (UoA), Medellin, Colombia; 31 were *PSEN1* E280A mutation carriers (mean age = 37.16 SD = 6.06, 18 females) and 36 were non-carrier family members (mean age = 35.25 SD = 5.85, 19 female) matched by age, sex and education (Table 1). 25/31 carriers were cognitively unimpaired, as defined by a Clinical Dementia Rating = 0 and Functional Assessment Staging Scale (FAST) score ≤2. Six of 31 carriers had MCI, as defined by Clinical Dementia Rating = 0.5 and FAST = 3. One carrier did not have PET amyloid data. The study was approved by the Institutional Review Board Committees of UoA and the Massachusetts General Hospital (MGH, Boston, USA). All subjects gave signed informed consent before participating and were compensated. Researchers and participants were blind to genetic status.

**Table 1:** Demographic and cognitive data for non-demented carriers and non-carrier family members.

Demographics and cognition	**Non-carriers *n* = 36**	**Carriers *n* = 31**	***P*-value**
Age	35.25 (5.85)	37.16 (6.06)	0.19
Sex F (%)	19 (53%)	18 (58%)	0.59
Education	10.89 (4.19)	9.10 (4.11)	0.08
MMSE	28.97 (0.91)	27.29 (2.73)	0.002
CERAD word list delayed recall	7.67 (1.24)	5.45 (2.93)	0.0004

Groups were matched for age, sex ratio (Chi-Square), and education years (Independent *t*-test). Carriers had significantly lower MMSE and lower recall performance. CERAD, Consortium to Establish a Registry for Alzheimer’s Disease; MMSE, Mini-Mental State Examination.

### Procedure

Neuropsychological testing was completed at UoA within six months prior to their MGH visit for MR and PET imaging. Neuropsychological testing included the Colombian-adapted battery of the Consortium to Establish a Registry for Alzheimer’s Disease. Of interest, testing included the Mini-Mental State Examination (MMSE) and the Consortium to Establish a Registry for Alzheimer’s Disease word-list learning task with delayed (20 min) recall. The MMSE was included in analysis as a measure of global cognition and delayed recall was included as this has been shown to be the most sensitive cognitive metric to date for this population.^22^

### Neuroimaging data

#### MRI

**Figure 1:**
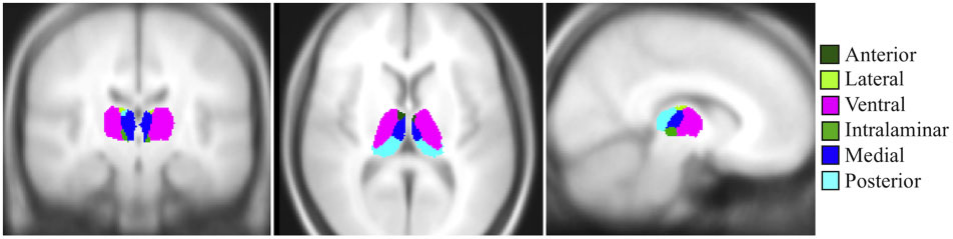
**Visualization of the six major thalamic subregions in MNI space.** See Table 1 for a list of thalamic nuclei included in each subregion.

All participants underwent an anatomical MRI scan at the MGH Athinoula A. Martinos Center for Biomedical Imaging using a Siemens Tim Trio 3T system with a 12-channel phased array head coil. Anatomical MRI scans were acquired using a T1-weighted magnetization-prepared rapid gradient echo sequence. A development version of FreeSurfer was used to automatically segment the thalamus based on a probabilistic atlas.^23^ This tool produces data for the whole thalamus and for 25 thalamic nuclei (see Table 2 and Fig. 1), which can be grouped into six bilateral subregional thalamic volumes, producing six regions of interest (ROIs)^20^: anterior, lateral, ventral, intralaminar, medial and posterior.

**Table 2:** Thalamic nuclei included in each subregion.

Thalamic nuclei included in each subregion	
Anterior	anteroventral
	anterodorsal
Lateral	laterodorsal
	lateral posterior
Ventral	ventral anterior
	ventral anterior magnocellular
	ventral lateral anterior
	ventral lateral posterior
	ventral posterolateral
	ventromedial
Intralaminar	central medial
	central lateral
	paracentral
	centromedian
	parafascicular
Medial	paratenial
	reuniens (medial ventral)
	mediodorsal medial magnocellular
	mediodorsal lateral parvocellular
Posterior	lateral geniculate
	medial geniculate
	limitans (suprageniculate)
	pulvinar anterior
	pulvinar medial
	pulvinar lateral
	pulvinar inferior

Thalamic nuclei (right side) included in each subregion (left). The thalamic parcellation tool used in the current study (Iglesias et al. 2018) provides 25 thalamic nuclei which were grouped into six larger subregions.

#### PET

PET images were acquired within the same week as MRI. For amyloid, we used 11C Pittsburgh Compound B (PiB), as described previously.^24^ For tau, we used 18F flortaucipir (FTP), prepared at MGH and validated for human use.^25^ For PiB, the values used for comparison were the distribution volume ratio (DVR) using cerebellar grey as a reference tissue; regional time-activity curves were used to compute regional DVRs for each ROI using the Logan graphical method applied to data from 40 to 60 min after injection. FTP specific binding was expressed in FreeSurfer ROIs as the standardized uptake value ratio to cerebellum. PiB retention was assessed using an aggregate cortical ROI, which included frontal, lateral-temporal and retrosplenial cortices, as well as striatal and thalamic ROIs.^26^^,^^27^ The two ROIs for tau binding comprised the bilateral entorhinal cortex (EC), as the first location of tau buildup and the inferior temporal cortex, as it represents the best proxy of early tau spreading to neocortex.^28^

### Statistical analysis

All statistical analyses were conducted using SPSS Statistics, version 26.0 (Armonk, NY: IBM Corp.). Independent *t*-tests were used to compare age, education years, and cognitive performance between carriers and non-carriers, and a chi-square test to compare sex distribution. A general linear model was used to assess group differences in brain volumes, with group as fixed factor and sex, education, and estimated total intracranial volume (ICV) as covariates. Partial Pearson correlations with sex, education and ICV as covariates were used to assess associations between thalamic volumes and age, PET ROIs, and cognitive measures. PET data were not partial-volume corrected. A family-wise alpha of 0.05 was used. Partial-eta^2^ for general linear model effect sizes were reported.

### Data availability

Anonymized clinical, genetic and imaging data are available upon request, subject to an internal review by Y.T.Q. and F.L. to ensure that the participants’ anonymity, confidentiality, and *PSEN1* E280A carrier or non-carrier status are protected. Data requests will be considered based on a proposal review, and completion of a data sharing agreement, in accordance with the University of Antioquia and MGH institutional guidelines. Please submit data requests to Y.T.Q. (yquiroz@mgh.harvard.edu).

## Results

### Group differences in subregional thalamic volumes

**Figure 2:**
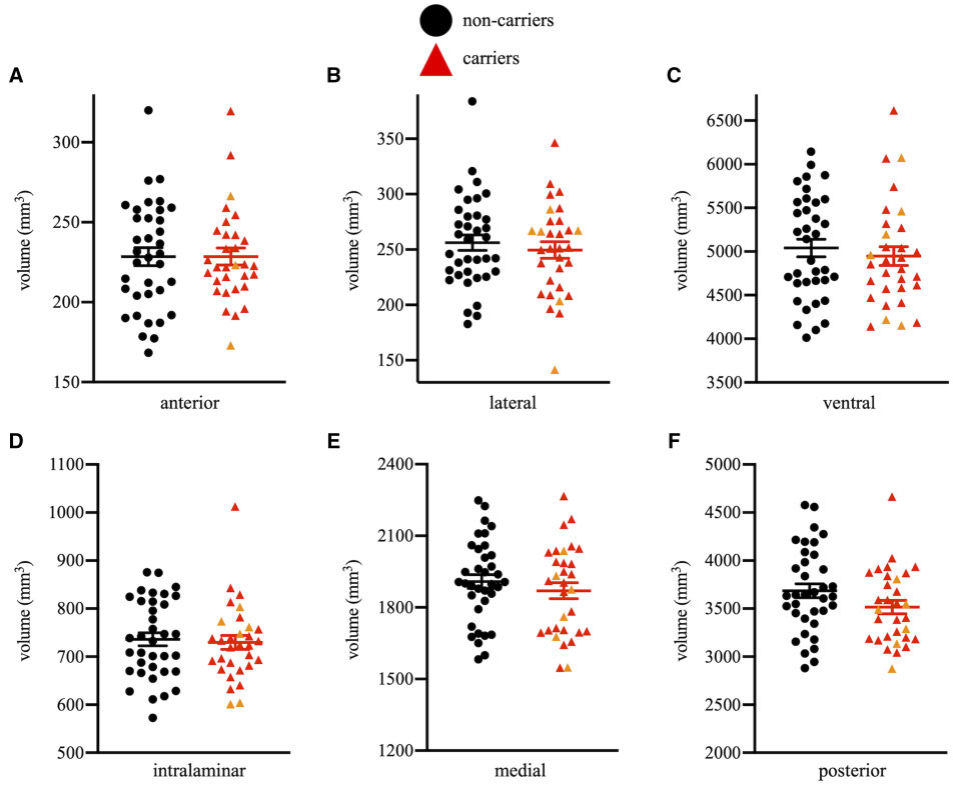
**Group scatter plots showing volumes for the six subregional thalamic volumes.** (**A**) anterior, (**B**) lateral, (**C**) ventral, (**D**) intralaminar, (**E**) medial and (**F**) posterior. No significant differences were found between groups. Asymptomatic carriers are shown in red triangles and non-carriers are shown in black circles. MCI carriers are shown in orange triangles for visual purposes only.

No significant differences between mutation carriers and non-carriers were found in any of the six subregional thalamic volumes or the whole thalamus volume [*F*’s(1,62) < 2.34, *P*’s > 0.13, partial-eta^2^’s > 0.04; Fig. 2].

### Associations between subregional thalamic volumes and age

**Figure 3:**
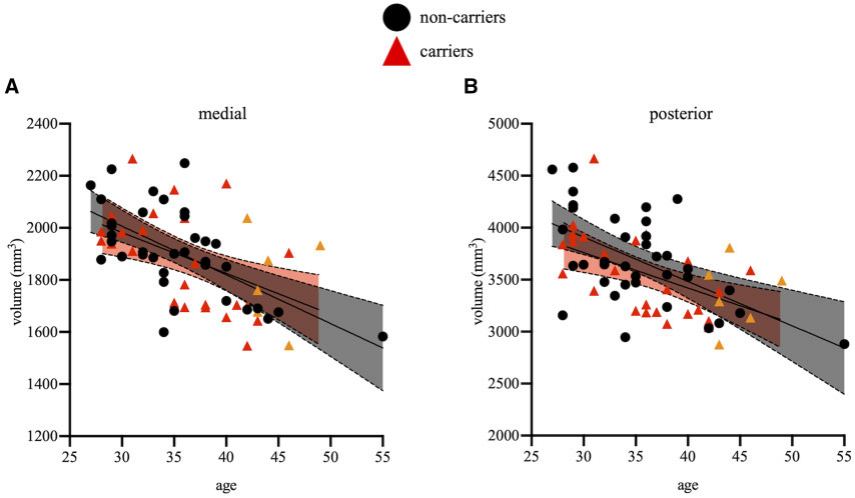
**Scatterplots showing associations between age and subregional thalamic volumes.** After controlling for sex, education and ICV, increasing age was related to lower medial and posterior volumes, but only significantly so in non-carriers. Asymptomatic carriers are shown in red triangles and non-carriers are shown in black circles. MCI carriers are shown in orange triangles for visual purposes only. Shaded areas represent 95% confidence intervals.

Partial Pearson correlations, controlling for sex, education and ICV, showed that, in the whole sample, increasing age was related to lower volumes of medial (*r* = −0.37, *P* = 0.003) and posterior thalamic subregions (*r* = −0.36, *P* = 0.003), as well as whole thalamus volume (*r* = −0.28, *P* = 0.03). In carriers, no correlations reached statistical significance (*r*’s < |0.33|, *P*’s > 0.09, Fig. 3). In non-carriers, increasing age was significantly associated with lower volumes of the medial subregion (*r* = −0.48, *P* = 0.005), the posterior subregion (*r* = −0.38, *P* = 0.03) and the whole thalamus (*r* = −0.41, *P* = 0.02). All associations between age and other subregional thalamic volumes in either group were not significant (*r*’s < |0.20|, *P*’s > 0.27; see Supplementary Table 1).

### Associations between subregional thalamic volumes and Alzheimer’s disease-related neuropathology

**Figure 4:**
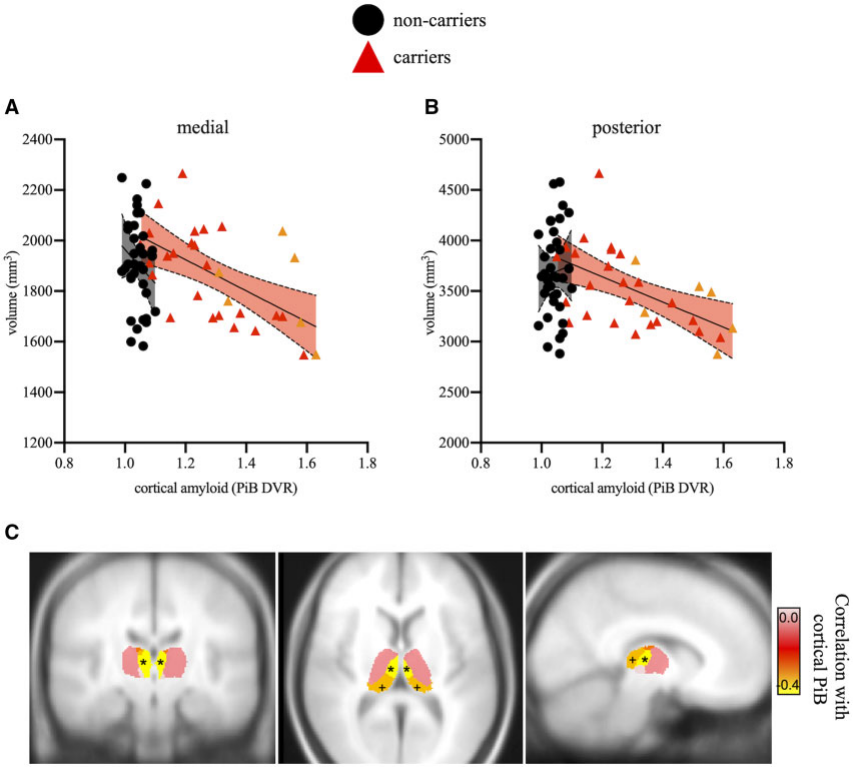
**Scatterplots showing associations between subregional thalamic volumes and PET-measured cortical amyloid burden.** After controlling for sex, education and ICV, higher cortical amyloid levels were correlated with lower volumes of medial (**A**) and, to a lesser extent, posterior (**B**) thalamic subregions. Asymptomatic carriers are shown in red triangles and non-carriers are shown in black circles. MCI carriers are shown in orange triangles for visual purposes only. Shaded areas represent 95% confidence intervals. (**C**) Visualization of correlations in MNI space. DVR, distribution volume ratio; PiB, Pittsburg Compound-B. *P = 0.03; +*P* = 0.07.

In carriers, higher levels of cortical amyloid were associated with lower volumes of medial (*r* = −0.41, *P* = 0.03; Fig. 4A) and posterior thalamic subregions (*r* = −0.35, *P* = 0.07, Fig. 4B), albeit the latter correlation was only trending towards significance. Because evidence of striatal and thalamic amyloid burden has been shown in early ADAD,^26^ we also examined whether thalamic subregional volumes were related to regional amyloid in these structures. Higher levels of striatal amyloid were associated with lower volumes of medial (*r* = −0.45, *P* = 0.02) and posterior thalamic subregions (*r* = −0.51, *P* = 0.007). In contrast, amyloid burden in the whole thalamus was only significantly correlated with lower posterior thalamic subregional volume (*r* = −0.42, *P* = 0.03). Although the PiB DVR range was quite restricted and the values were below pathological levels (0.99–1.10), a significant negative correlation was observed between the medial subregional volume and cortical amyloid for non-carriers (*r* = −0.35, *P* = 0.04). Importantly, no correlations were observed for striatal or thalamic amyloid for non-carriers (*r*’s < |0.22|, *P*’s > 0.22). No other correlations were significant between neuropathology and thalamic subregions (*r*’s < |0.30|, *P*’s > 0.13).

**Figure 5:**
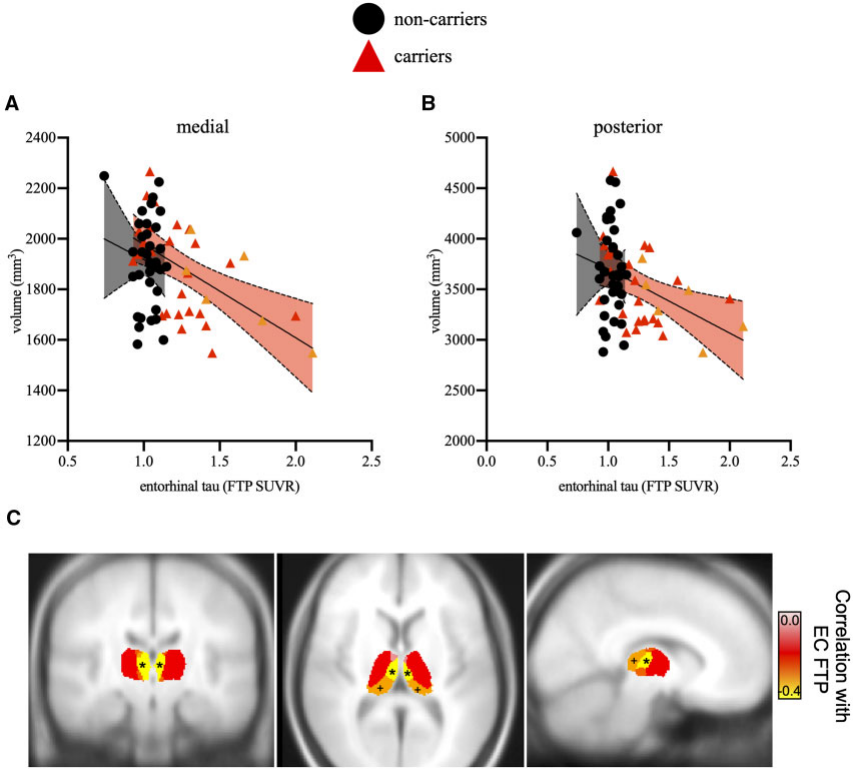
**Scatterplots showing associations between subregional thalamic volumes and PET-measured entorhinal (EC) tau burden.** After controlling for sex, education and ICV, higher entorhinal tau levels were correlated with lower volumes of medial (**A**) and, to a lesser extent, posterior (**B**) thalamic subregions. Asymptomatic carriers are shown in red triangles and non-carriers are shown in black circles. MCI carriers are shown in orange triangles for visual purposes only. Shaded areas represent 95% confidence intervals. (**C**) Visualization of correlations in MNI space. FTP, flortaucipir; SUVR, standardized uptake volume ratio. *P = 0.009; +*P* = 0.08.

Higher levels of EC tau were similarly related to lower volumes of medial (*r* = −0.48, *P* = 0.009, Fig. 5A) and, marginally to posterior thalamic subregions (*r* = −0.33, *P* = 0.08, Fig. 5B). Increased EC tau was marginally related to decreased whole thalamus volume (*r* = −0.36, *P* = 0.06). No other significant correlations were found between EC tau and other subregional thalamic volumes (*r*’s < |0.29|, *P*’s > 0.14). No associations were found between inferiotemporal tau burden and any of the thalamic subregions or whole thalamic volume (*r*’s < |0.32|, *P*’s > 0.09). In non-carriers, no associations were found between regional tau levels and thalamic subregional volumes (*r*’s < |0.29|, *P* > 0.10).

### Associations between subregional thalamic volumes and cognitive measures

No correlations were found between any of the thalamic subregions and verbal memory (*r*’s < |0.31|, *P*’s > 0.11) or MMSE scores (*r*’s < |0.24|, *P*’s > 0.18), regardless of group.

## Discussion

Subregions of the thalamus are differentially affected by Alzheimer’s disease neuropathology,^5^^,^^6^ but whether and how this translates to *in vivo* neurodegeneration in preclinical/prodromal stages remains unclear. In this study, we leveraged data from the world’s largest ADAD kindred due to a single mutation to investigate whether subregional thalamic volumes were affected in non-demented carriers who will go on to develop dementia. We also investigated the associations between subregional thalamic volumes, PET-measured amyloid-beta and tau burden, and cognitive performance.

At the group level, no volumetric differences were found between carriers and non-carriers. Medial and, to a lesser extent, posterior subregional volumes of the thalamus seem to be linked to early clinical progression, as suggested by their association with early Alzheimer’s disease pathology burden (cortical and striatal amyloid as well as EC tau). To the best of our knowledge, this is the first study to evaluate the thalamus, and its relation to *in vivo* neuropathology, as a complex, heterogenous structure in ADAD.

The lack of group differences in subregional and whole thalamic volumes in the current study supports recent findings. In a recent study, no volumetric differences in thalamic subregions were reported between individuals at prodromal stages of sporadic Alzheimer’s disease (i.e. amyloid-positive MCI) and cognitively healthy individuals.^20^ In contrast, this pattern does not confirm our previous findings with a different sample of *PSEN1* E280A mutation carriers^18^ or findings from other ADAD studies,^14–16^ in which reduced (whole) thalamic volume was found in mutation carriers. This might be explained by the inclusion of carriers with different age ranges and different stages of disease, or carriers of heterogenous mutations in previous studies. A recent study found that different pathogenic *PSEN1* mutations affect the amyloid-beta_42/40_ ratio in distinctive ways. That is, some mutations stopped the production of both forms of the peptide altogether while others increased or decreased the ratio.^29^ Another group, using the DIAN dataset, found that different genotypes showed greater and earlier accumulation of amyloid-beta in the cortex compared to the striatum, while others showed the opposite pattern.^30^ Another possible explanation for conflicts with previous work stems from using different volumetric segmentation and analyses, including tensor- and voxel-based morphometry. Altogether, the current study reinforces the idea that previous conflicting findings might be partially explained by the fact that the thalamus was considered a single, homogeneous structure.

In the current study, lower volume of medial and, to a lesser extent, posterior thalamic subregions were related to greater levels of cortical amyloid and entorhinal tau burden (Figs. 4 and 5), the earliest site of observable tau pathology in vivo. Higher striatal amyloid burden was also associated with lower medial and posterior subregional thalamic volumes, and we also observed a correlation between thalamic amyloid burden and posterior subregional thalamic volume. These results support earlier findings in which striatal and thalamic amyloid was found early in ADAD (e.g. see Refs.^15^^,^^16^^,^^27^^,^^31^). Of note, these associations are independent of the role of sex, education, and ICV. This pattern suggests that thalamic atrophy in this ADAD kindred is both specific to Alzheimer’s disease neuropathology and specific to certain thalamic subregions.

The medial thalamus, particularly the mediodorsal nucleus, is known to play a role in cognitive function.^32–34^ A recent study suggests a crucial role of medial nuclei in moderating the control of whole-brain cortical networks during complex cognitive performance.^34^ In contrast, posterior thalamic groups (i.e. pulvinar and lateral geniculate nucleus) are more involved in lower-level functions, such as visuospatial processing^35^ and sleep-wake regulation.^4^ The pulvinar presents neuronal phase advancement to the cortex at sleep onset.^4^ The midline group also play a crucial role in sleep maintenance by regulating cortico-thalamic oscillations.^36^ Synaptic and neuronal loss in these regions may explain sleep disturbances observed in Alzheimer’s disease, which are thought to begin in preclinical stages.^37^ In sum, nuclei-specific thalamic pathology might help explain both cognitive and sleep-related dysfunction in Alzheimer’s disease.

Early histopathological work that described the anterior thalamus (anterodorsal nucleus specifically) to be highly affected by Alzheimer’s disease pathology^5^^,^^6^ as well as its known role in memory^3^ could predict a role of the anterior subregional thalamic volume in the current study, but this was not the case. A plausible explanation may arise from the possibility that anterior nuclei are affected later in the disease, as suggested by a recent study in which anterior thalamic atrophy was only observed in patients with Alzheimer’s disease when compared to amyloid-positive MCI and healthy controls, but no such differences were observed between MCIs and controls.^20^ The current study assessed the role of thalamic subregional volumes in preclinical and prodromal Alzheimer’s disease, as most of the mutation carriers were cognitively healthy.

The current study has several limitations. Our small sample size likely limited our ability to detect some significant effects, meaning that null findings, including those trending toward significance, may be significant with greater power. Although we restricted the number of statistical comparisons, our findings should be interpreted with caution due to the potential issue of multiple comparisons. Future studies should examine larger samples, with a broader range of age, as well as longitudinal follow-up. Furthermore, we limited our analyses to six aggregate subregional volumes of the thalamus to minimize false positives. The thalamus parcellation tool utilized here segments the thalamus into 25 unilateral nuclei. Future studies with larger samples using similar parcellation tools may consider specific thalamic nuclei instead of aggregate subregions, other segmentation tools, and higher resolution neuroimaging.

In this study, we investigated volumetric changes in thalamic subregions and their associations with Alzheimer’s disease-related neuropathology and cognition, from preclinical to prodromal stages of ADAD. Investigating preclinical stages of sporadic Alzheimer’s disease, which depends on clinical diagnosis (i.e. amnestic MCI) or, at best, biomarker-defined increased risk (i.e. amyloid-positivity), might result in ambiguous findings since these individuals might never develop dementia due to Alzheimer’s disease. ADAD allows researchers to study disease progression from preclinical stages in individuals that are virtually destined to develop early-onset Alzheimer’s disease while simultaneously removing comorbidities related to ageing and other pathologies (e.g. cardiovascular injury). We found that medial and, to a lesser extent, posterior subregions of the thalamus were related to disease pathology in ADAD, supporting the notion that the thalamus is differentially affected by the disease and cannot be considered a unitary structure in future research.

## Supplementary material

Supplementary material is available at *Brain Communications* online.

## Supplementary Material

fcab101_Supplementary_Data

## Abbreviations

ADAD =: autosomal-dominant Alzheimer’s disease
CERAD =: Consortium to Establish a Registry for Alzheimer’s Disease
DVR =: distribution volume ratio
EC =: entorhinal cortex
FTP =: flortaucipir (18F-AV-1451; 18F-T807)
MCI =: mild cognitive impairment
MMSE =: Mini-Mental State Examination
PiB =: Pittsburgh Compound B
PSEN1 =: Presenilin-1
ROI =: region of interest
