# Supplementary material for: Associations between subregional thalamic volume and brain pathology in autosomal dominant Alzheimer’s disease
Source: Brain Commun. 2021 May 10;3(2):fcab101. doi: 10.1093/braincomms/fcab101 (PMC8172494; doi:10.1093/braincomms/fcab101)
Supplement: fcab101_Supplementary_Data [file fcab101_supplementary_data.docx]

Supplementary Table 1. Partial Pearson correlations, with covariates sex, years of education, and estimated total intracranial volume

| *Partial correlations for non-carriers*  *Covariates: sex, education years, intracranial volume* | | | | | | | | |
| --- | --- | --- | --- | --- | --- | --- | --- | --- |
|  |  | anterior | lateral | ventral | intralaminar | medial | posterior | whole thalamus |
|  |  |  |  |  |  |  |  |  |
| age | r | -0.15 | 0.00 | -0.20 | -0.16 | **-0.48** | **-0.38** | **-0.41** |
|  | p | 0.41 | 1.00 | 0.27 | 0.38 | **0.01** | **0.03** | **0.02** |
|  |  |  |  |  |  |  |  |  |
|  |  |  |  |  |  |  |  |  |
| PET amyloid | r | 0.01 | -0.04 | -0.11 | -0.25 | **-0.35** | 0.05 | -0.14 |
| (cortical) | p | 0.97 | 0.84 | 0.53 | 0.16 | **0.04** | 0.78 | 0.44 |
|  |  |  |  |  |  |  |  |  |
| PET amyloid | r | 0.16 | 0.14 | 0.19 | 0.16 | -0.03 | 0.04 | 0.15 |
| (striatum) | p | 0.37 | 0.45 | 0.30 | 0.39 | 0.88 | 0.81 | 0.42 |
|  |  |  |  |  |  |  |  |  |
| PET amyloid | r | 0.19 | 0.04 | 0.24 | 0.15 | 0.14 | 0.07 | 0.22 |
| (thalamus) | p | 0.29 | 0.82 | 0.18 | 0.41 | 0.43 | 0.70 | 0.22 |
|  |  |  |  |  |  |  |  |  |
| PET tau | r | 0.08 | -0.06 | -0.18 | -0.13 | -0.09 | 0.01 | -0.13 |
| (entorhinal) | p | 0.67 | 0.76 | 0.31 | 0.47 | 0.61 | 0.96 | 0.48 |
|  |  |  |  |  |  |  |  |  |
| PET tau | r | -0.12 | -0.13 | -0.25 | -0.17 | -0.21 | -0.17 | -0.29 |
| (infertemporal) | p | 0.50 | 0.47 | 0.16 | 0.35 | 0.24 | 0.35 | 0.10 |
|  |  |  |  |  |  |  |  |  |
|  |  |  |  |  |  |  |  |  |
| verbal memory | r | 0.06 | 0.16 | -0.04 | -0.04 | 0.17 | -0.12 | -0.04 |
|  | p | 0.73 | 0.38 | 0.81 | 0.81 | 0.36 | 0.50 | 0.84 |
|  |  |  |  |  |  |  |  |  |
| MMSE | r | -0.15 | 0.01 | 0.06 | 0.01 | 0.24 | -0.09 | 0.04 |
|  | p | 0.39 | 0.94 | 0.75 | 0.98 | 0.18 | 0.64 | 0.83 |
|  |  |  |  |  |  |  |  |  |
| *Partial correlations for carriers*  *Covariates: sex, education years, intracranial volume* | | | | | | | | |
|  |  | anterior | lateral | ventral | intralaminar | medial | posterior | whole thalamus |
|  |  |  |  |  |  |  |  |  |
| age | r | 0.13 | -0.03 | 0.06 | -0.06 | -0.29 | -0.33 | -0.15 |
|  | p | 0.52 | 0.89 | 0.78 | 0.77 | 0.14 | 0.09 | 0.45 |
|  |  |  |  |  |  |  |  |  |
|  |  |  |  |  |  |  |  |  |
| PET amyloid | r | 0.08 | -0.30 | 0.08 | -0.02 | **-0.41** | -0.35 | -0.19 |
| (cortical) | p | 0.71 | 0.13 | 0.70 | 0.92 | **0.03** | 0.07 | 0.36 |
|  |  |  |  |  |  |  |  |  |
| PET amyloid | r | -0.03 | -0.24 | -0.13 | -0.19 | **-0.45** | **-0.51** | -0.37 |
| (striatum) | p | 0.87 | 0.23 | 0.51 | 0.33 | **0.02** | **0.01** | 0.06 |
|  |  |  |  |  |  |  |  |  |
| PET amyloid | r | -0.27 | -0.31 | -0.21 | -0.20 | -0.31 | **-0.42** | -0.36 |
| (thalamus) | p | 0.17 | 0.12 | 0.30 | 0.31 | 0.12 | **0.03** | 0.06 |
|  |  |  |  |  |  |  |  |  |
| PET tau | r | -0.07 | -0.28 | -0.19 | -0.29 | **-0.48** | -0.33 | -0.36 |
| (entorhinal) | p | 0.72 | 0.15 | 0.34 | 0.14 | **0.01** | 0.08 | 0.06 |
|  |  |  |  |  |  |  |  |  |
| PET tau | r | -0.08 | -0.32 | -0.03 | -0.20 | -0.32 | -0.13 | -0.17 |
| (inferiotemporal) | p | 0.71 | 0.09 | 0.86 | 0.30 | 0.10 | 0.51 | 0.39 |
|  |  |  |  |  |  |  |  |  |
|  |  |  |  |  |  |  |  |  |
| verbal memory | r | 0.06 | 0.13 | 0.16 | 0.31 | 0.31 | 0.30 | 0.29 |
|  | p | 0.78 | 0.50 | 0.43 | 0.11 | 0.11 | 0.12 | 0.14 |
|  |  |  |  |  |  |  |  |  |
| MMSE | r | 0.08 | 0.28 | -0.20 | 0.07 | 0.08 | 0.17 | 0.00 |
|  | p | 0.67 | 0.15 | 0.31 | 0.74 | 0.70 | 0.38 | 1.00 |
